# Efficacy and safety of valganciclovir in patients with symptomatic congenital cytomegalovirus disease: Study Protocol Clinical Trial (SPIRIT Compliant)

**DOI:** 10.1097/MD.0000000000019765

**Published:** 2020-04-24

**Authors:** Ichiro Morioka, Yasumasa Kakei, Takashi Omori, Kandai Nozu, Kazumichi Fujioka, Tetsushi Yoshikawa, Hiroyuki Moriuchi, Yoshinori Ito, Akira Oka

**Affiliations:** a Department of Pediatrics and Child Health, Nihon University School of Medicine; b Department of Oral and Maxillofacial Surgery; c Division of Biostatistics, Department of Social/Community Medicine and Health Science; d Department of Pediatrics, Kobe University Graduate School of Medicine, Kobe; e Department of Pediatrics, Fujita Health University School of Medicine, Toyoake; f Department of Pediatrics, Nagasaki University Graduate School of Medicine, Nagasaki; g Department of Pediatrics, Nagoya University Graduate School of Medicine, Nagoya; h Department of Pediatrics, The University of Tokyo, Tokyo, Japan.

**Keywords:** auditory brainstem response, clinical trial, congenital cytomegalovirus disease, valganciclovir, viral load

## Abstract

**Background:**

Congenital cytomegalovirus (CMV) disease, a common mother-to-child infection, can lead to neurological sequelae. Some clinical trials have shown that oral valganciclovir (VGCV) can improve hearing and neurodevelopmental impairment in infants with congenital CMV disease. However, VGCV has neither been approved in Japan nor other countries as a treatment for this disease by the government health insurance.

**Methods:**

This study is a non-randomized, prospective, open-label, multicenter, single-arm clinical trial and will include subjects meeting the following criteria: confirmation of positive CMV-DNA amplification in urine by an in vitro diagnostic test within 21 days of age; congenital CMV disease with one or more central nervous system disorders—microcephaly, hydrocephalus or ventricular enlargement, periventricular calcification, cortical hypoplasia or white matter injury, retinal choroiditis, and abnormal auditory brainstem response (ABR); and infants within 2 months of age with a gestational age ≥32 weeks at birth and weighing ≥1800 g at the time of registration. Subjects will be orally administered 16 mg/kg VGCV twice daily for 6 months. The target number of cases for enrollment between February 3, 2020 and July 31, 2021 is 25. Primary endpoint is the change in whole blood CMV loads before and after 6 months of treatment. The important secondary endpoint is the change in ABR (both best and total ear hearing assessments) before and after 6 months of treatment. The safety endpoints are adverse events and drug side effects.

**Discussion:**

To the best of our knowledge, this multicenter, open-label, single-arm study will be the first well-designed clinical trial to evaluate the efficacy of oral VGCV in infants with congenital CMV diseases. The findings will reveal the efficacy and safety of oral VGCV treatments and enable the approval of oral VGCV as a treatment for infants with congenital CMV disease by the government health insurance of Japan.

## 1 Introduction

Congenital cytomegalovirus (CMV) disease, a condition with a greater frequency than Down syndrome, neural tube defects, or fetal alcohol syndrome, is the most common cause of congenital central nervous system disorders in developed countries.^[1]^ According to a Japanese epidemiological survey, congenital CMV infection occurred in 1 in 300 births,^[2]^ 27% of which had sequelae of hearing difficulty, epilepsy, neurodevelopmental disorder, and developmental delay (82% of symptomatic infants with/without antiviral therapy and 12% of asymptomatic infants at birth).^[3]^ The disease burden associated with congenital CMV disease, especially in symptomatic infants at birth, is substantial in children in Japan.

Ganciclovir (GCV) is phosphorylated in CMV-infected cells and inhibits DNA replication by limiting viral DNA elongation. Valganciclovir (VGCV) is the l-valine ester form of GCV (prodrug), which is orally administered and rapidly converted to GCV by intestinal and hepatic esterase. Because its bioavailability is approximately 60%, not only can it be used as a maintenance treatment but also as an initial treatment for CMV infection.^[4,5]^ Earlier clinical trials in the United States have shown that treatment with intravenous GCV for 6 weeks beginning at the newborn period improved hearing and neurodevelopment in children with congenital CMV disease that involves the central nervous system.^[6,7]^ Consistent with these findings, a phase III clinical trial in the United States showed the efficacy and safety of oral VGCV treatment for 6 months beginning at the newborn period.^[8]^ In Japan, the efficacy for hearing, psychomotor development, and safety of oral VGCV treatment for 6 weeks or 6 months has also been reported in infants with congenital CMV disease that involved the central nervous system.^[9–11]^

Since January 2018, the urine CMV-DNA amplification test for infants at risk of congenital CMV infection and are within 3 weeks of age has become available for a definitive diagnosis under the government health insurance coverage in Japan.^[4]^ Presently, VGCV treatment is covered by the Japanese government health insurance for patients with the following conditions: CMV disease in acquired immunodeficiency syndrome, organ transplantation including hematopoietic stem cell transplantation, and malignant tumors, or onset suppression of CMV infection in organ transplantation excluding hematopoietic stem cell transplantation. However, VGCV treatment for congenital CMV disease is not approved by the government health insurance not only in Japan but also worldwide. Because congenital CMV is a progressive disease with no other effective treatments and VGCV remains an off-label drug for its treatment, in Japan, oral VGCV treatment is currently being performed at the discretion of the attending pediatrician and neonatologist with institution review board (IRB) approval and a well-informed consent from the parents.

Therefore, a phase III, multicenter, open-label, single-arm clinical trial of oral VGCV treatment for 6 months will be conducted in infants with congenital CMV disease that involves the central nervous system and are within 2 months of age to enable a high frequency of any sequelae for approval by the government health insurance in Japan. The objective of this clinical trial is to determine the efficacy and safety of oral VGCV treatments in infants with congenital CMV disease.

## 2 Methods

### 2.1 Study design/setting

**Figure 1:**
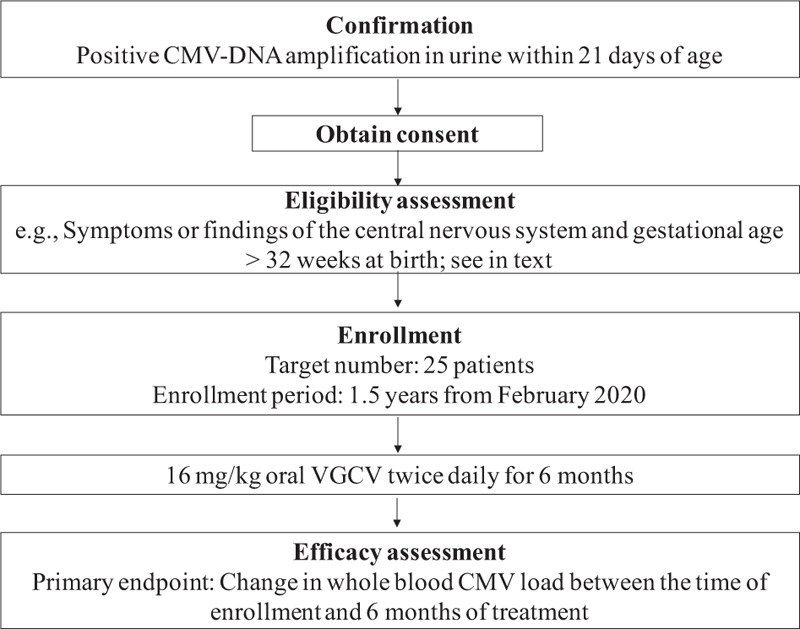
Summary of the study protocol. CMV = cytomegalovirus, VGCV = valganciclovir.

This study is a non-randomized, prospective, open-label, multicenter clinical trial that commenced on February 3, 2020. The expected date of completion (last visit of last patient) is end of February 2022. A summary of the study is presented in Fig. 1. This study will be performed at 6 academic hospitals, namely, The University of Tokyo Hospital, Nihon University Itabashi Hospital, Nagoya University Hospital, Fujita Medical University Hospital, Kobe University Hospital, and Nagasaki University Hospital in Japan. The analysis period will continue for 1 year after the day of registration. This study protocol follows the SPIRIT-statement. All study data will be stored and archived in the data center at EP-CRSU Co., Ltd, Tokyo, Japan.

### 2.2 Study population

#### 2.2.1 Inclusion criteria

(1) Confirmation of positive CMV-DNA amplification in urine by an in vitro diagnostic test within 21 days of age.
(2) Congenital CMV disease with one or more of the following central nervous system disorders:
  1) Microcephaly
  2) Hydrocephalus or ventricular enlargement
  3) Periventricular calcification
  4) Cortical hypoplasia or white matter injury
  5) Retinal choroiditis
  6) Abnormal auditory brainstem response (ABR).
(3) <60 days of age at informed consent.
(4) Gestational age >32 weeks at birth.
(5) Body weight at study enrollment >1800 g.
(6) Signed informed consent from parents or legal guardians.

#### 2.2.2 Exclusion criteria

(1) Patients with bacterial infection requiring antibiotics at the time of study enrollment.
(2) Renal insufficiency (serum creatinine level >1.5 mg/dL) at the time of study enrollment.
(3) Encephalopathy and hydrocephalus owing to other causes.
(4) Neutrophil count <500/mm^3^ or platelet count <25,000/mm^3^.
(5) Infants born to women with human immunodeficiency virus (HIV) or infants with HIV.
(6) Patients deemed inappropriate by a study investigator or sub-investigators.

### 2.3 Intervention

After the screening for patient registration in this study, the subjects will be orally administered 16 mg/kg VGCV twice daily for 6 months in the treatment period. CMV loads in the whole blood and urine will be measured every week until 6 weeks after treatment initiation and every month thereafter. A summary of other study outcomes, assessments, and procedures for subjects in this study is presented in Table 1. GCV, foscarnet, and letermovir are prohibited during this clinical trial.

**Table 1:**
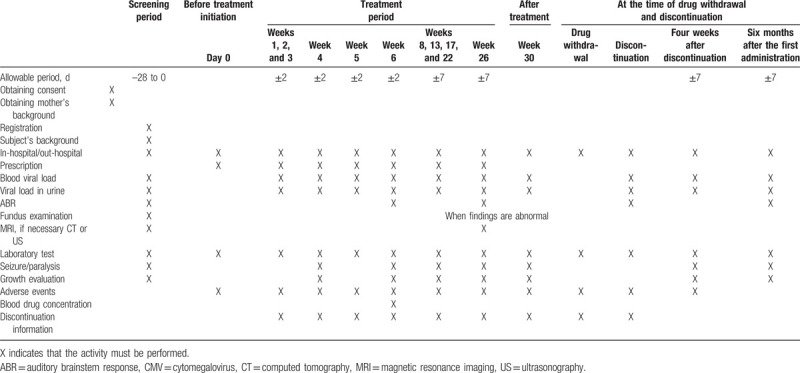
Summary of the study assessments and procedures.

### 2.4 Outcomes

The primary endpoint for efficacy:

- Change in whole blood CMV load before and after 6 months of treatment.

The secondary endpoint for efficacy:

- Change in ABR (both best and total ear hearing assessments) before and after 6 months of treatment was set as the important secondary endpoint.

Other secondary endpoints:

(1) Dynamics of CMV load in whole blood during the study period.
(2) Change in plasma CMV load before and after 6 months of treatment.
(3) Dynamics of CMV load in urine during the study period.
(4) Improvement of thrombocytopenia, liver function disorder, and retinitis.
(5) Growth indicators, such as height, weight, head circumference, and ability to hold up the head and roll over.

The secondary endpoints for safety:

(1) Adverse events.
(2) Adverse drug reactions.

### 2.5 Adverse events

In our study, an adverse event will be defined as any disease, disability, death, or infection that occurs during the study. The investigator or sub-investigator will record all adverse events in the case report form (CRF), treat, and follow-up with the patient until resolution is achieved during the study. If the investigator or sub-investigator recognizes a potentially causal relationship to the study drug, all adverse reactions will be recorded and reported to the review board. The criteria for dose adjustment and drug withdrawal were set according to a phase III clinical trial by Kimberlin et al.^[8]^ If the neutrophil count falls below 500/mm^3^, the study drug administration is discontinued until the neutrophil count reaches ≥750/mm^3^. If the neutrophil count falls again below 750/mm^3^, the dose is reduced to 50%. If the neutrophil count falls again below 500/mm^3^ by the drug with the reduced dose, the study drug administration is discontinued. If the platelet count falls below 50,000/mm^3^, the study drug administration is discontinued until the platelet count reaches 50,000/mm^3^ or higher. If the hemoglobin level falls below 8 g/L, the study drug administration is discontinued until the hemoglobin level reaches ≥8 g/dL. If serum aspartate aminotransferase or alanine aminotransferase level is >10 times from the baseline or >500 U/L, the study drug administration is discontinued until it reaches <10 times from the baseline or <500 U/L.

### 2.6 Data collection and management

The investigator or sub-investigator will enter the CRF data for each subject into the electronic data capture (EDC) system. The investigator will confirm that the entered CRF data is complete and correct, electronically sign the CRF on the EDC system, and generate a printout of the signed CRF for filing. The CRF printout will be retained. If any queries arise regarding the CRF data entered by the staff at the data center, the primary investigator or sub-investigator will promptly respond to the queries.

### 2.7 Statistics

#### 2.7.1 Sample size calculation

The target number of subjects is 25. This sample size for the study was derived according to the proportion of non-worsened hearing for the important secondary endpoint (i.e., the change in ABR [best ear hearing assessments] before and after 6 months of treatment). According to Kimberlin et al,^[8]^ the proportion of non-worsened hearing after 6 months of VGCV administration was 88% (38/43). Additionally, they reported 59% (10/17) as the proportion for no treatment.^[6]^ When the expected value for the proportion of 88% and the proportion for no treatment of 59% are set, the number of subjects required for the statistical test of 1 sample proportion with a significance level of 0.05 and power of 0.8 is 19. After considering the expected dropout, the number of required subjects was set as 25.

For the primary endpoint, which is the change in whole blood CMV load before and after 6 months of treatment, a time plot of the viral load reported by Kimberlin et al^[8]^ could be constructed. From the figure,^[8]^ the change in whole blood CMV load before and after 6 months of treatment was approximately 1.6, and the standard error was approximately 0.25. These statistics reveal that the above targeted sample size is sufficient for detection of the primary endpoint in this study.

#### 2.7.2 Analysis population

The analysis populations for efficacy are the full analysis set (FAS) and the per protocol set (PPS). The FAS will also serve as the analysis population for safety. The FAS is defined as all subjects registered in this study and administered at least 1 dose of VGCV. The PPS is defined as subjects in the FAS, excluding those with any of the following significant protocol violation regarding study method and concomitant therapy: violation of the inclusion or exclusion criteria and critical violation of the protocol that could affect the efficacy evaluation. All analysis will be carried out with FAS; however, the primary endpoint and secondary endpoint will be analyzed with both FAS and PPS.

#### 2.7.3 Analysis

The handling of the enrolled subjects for analyses will be determined via a discussion among the coordinating investigators, committee, and biostatistician before data lock. If values are missing, they will not be interpolated for analysis of the primary endpoint; however, values at 6 weeks will be interpolated for analysis of the important secondary endpoint.

#### 2.7.4 Primary analysis for the primary endpoint

Median and 95% confidence interval for the change in whole blood CMV load before and after treatment for 6 months will be estimated. The number of subjects, proportion, and 95% confidence interval for the disappearance of whole blood CMV load will also be estimated. Wilcoxon signed-rank test with significance level of 0.05 (both sides) will be performed under the null hypothesis that the location parameter of the distribution of the change in whole blood CMV load before and after 6 months of treatment is 0.

#### 2.7.5 Analysis for the important secondary endpoint

According to the change in ABR (both best and total ear hearing assessments), we will conduct the following analysis based on the 4 categories before and after treatment for 6 months: (a) improved hearing,(b) no change–normal hearing,(c) no change–same degree of hearing, and (d) worsened hearing.

(1) For the best ear assessment with ABR, the proportion and 95% confidence interval of (a) + (b) + (c) will be estimated. According to the closed testing procedure, a statistical test of one population with a significance level of 0.05 (both sides) will only be applied when the above statistical test of the primary endpoint for the best ear assessment is significant. The null hypothesis of the statistical test states that this proportion should be ≤59%.
(2) For the total ear assessment with ABR, the proportion and 95% confidence interval of (a) + (b) + (c) will be estimated.
(3) For the best ear assessment with ABR, the proportion and 95% confidence interval of (a) + (b) interval will be estimated.
(4) For the total ear assessment with ABR, the proportion and 95% confidence interval of (a) + (b) interval will be estimated.

The thresholds for hearing are defined as follows: normal hearing, 0 to 20 dB; mild hearing abnormality, 21 to 45 dB; moderate hearing abnormality, 46 to 70 dB; and severe hearing abnormality, ≥71 dB.

#### 2.7.6 Analysis for other secondary endpoints

(1) Dynamics of CMV load in whole blood during the study period
  Whole blood CMV load at each time point for each subject will be plotted as a figure. A time plot of mean with standard deviation and size will be generated.
(2) Change in plasma CMV load before and after treatment for 6 months
  Number of subjects, proportion, and 95% confidence interval for the disappearance of plasma CMV load will be estimated.
(3) Dynamics of CMV load in urine during the study period
  CMV load in the urine at each time point for each subject will be plotted as a figure. A time plot of mean with standard deviation and size will also be generated.
(4) Improvement of thrombocytopenia, liver function disorder, and retinitis before and after treatment for 6 months
  Number of subjects, proportion, and 95% confidence interval for each improvement will be estimated.
(5) Growth indicators, such as height, weight, head circumference, and ability to hold the head up and roll over at each time point
  Summary statistics at each time point will be calculated. The proportion and 95% confidence interval to hold up the head and roll over at each time point will be estimated.

#### 2.7.7 Analysis for safety endpoints

The proportion of adverse events and adverse drug reactions will be calculated. A time plot of the values obtained from the laboratory test for each subject will be generated. The summary statistics of the laboratory test at each time point will also be calculated. The adverse event and adverse drug reaction in each patient will be listed by organ, and the details of each adverse drug reaction will be described in the list. Abnormal values of laboratory test, such as predefined or clinically important, will be presented in the list.

#### 2.7.8 Analysis for drug blood concentration

In this study, drug blood concentration at 6 months will be measured after VGCV administration. Because this drug is metabolized to GCV shortly after dosing, the blood concentration of GCV will also be measured. The measured blood concentration of GCV for each subject and the size, mean, standard deviation, maximum value, minimum value, and median will be reported. A scatter plot of the blood concentration of GCV with whole blood CMV load at 6 months will also be generated.

### 2.8 Monitoring and auditing

Periodic monitoring of the study will be performed to verify that the human rights and welfare of subjects are protected. The study will be safely conducted in accordance with the protocol and the applicable regulatory requirements under the good clinical practice (GCP), and data collection will be properly executed. The coordinating investigator will appoint monitors for the study. The items to be checked at monitoring are specified in the “Written procedure for implementation of study monitoring.”

For quality assurance, the study will be examined to ensure it abides by the protocol and written procedures, independently and separately from the routine activities of monitoring and quality control. The coordinating investigator will complete the “Written procedure auditing” form and will ensure that the appointed auditor scrutinizes the study in accordance with the “Written procedure for auditing.” The auditor will report the audit results to the coordinating investigator and the investigator at the site selected for the audit.

### 2.9 Ethics and trial status

The study is in compliance with the principles of the Declaration of Helsinki (1996), the principles of GCP, and all applicable regulatory requirements. This clinical trial was registered in the Japan Registry of Clinical Trials under the number, jRCT2051190075, on November 15, 2019. The study protocol was first authorized by the institutional review committee on October 24, 2019 at the Kobe University Graduate School of Medicine. Participant recruitment began on February 3, 2020. The expected date of completion (last visit of last patient) is the end of July 2021. Ethical approval was obtained from the IRB of Kobe University (ref approval no. 190025). Recruitment at other centers began after local ethical approval was obtained for the trial (approved on December 23, 2019 at The University of Tokyo Hospital, December 24, 2019 at Nihon University Itabashi Hospital, December 25, 2019 at Fujita Medical University Hospital, January 20, 2020 at Nagoya University Hospital, and January 28, 2020 at Nagasaki University Hospital).

Written informed consent will be obtained from all participants prior to the start of any study procedure. The parent(s) of the participant will be able to review the participant consent form (PCF) and confirm that they fully understand the details of the study procedures. Informed consent will be administered by a suitably qualified and experienced individual who has been allocated this duty by the investigator.

The PCF was approved by the IRB of Kobe University to inform the families of participants that the findings of the trial will be submitted for publication in a scientific journal. Participants will not be identifiable in any publication. The results will be disseminated via presentations to the community and publication in a peer-reviewed journal.

## 3 Discussion

VGCV treatment for congenital CMV disease has not been approved by government health insurance worldwide. However, the International Congenital Cytomegalovirus Conference has recommended the administration of 16 mg/kg of VGCV twice daily for 6 months for the treatment of moderate to severe symptomatic congenital CMV infection at birth^[12]^ owing to the following findings. First, a phase III, randomized, double-blind, placebo-control clinical trial for congenital CMV disease involving the central nervous system showed that the intravenous administration of 6 mg/kg GCV twice daily for 6 weeks significantly improved hearing and neurodevelopmental status of patients.^[6,7]^ Additionally, the common adverse event in patients treated with GCV was found to be neutropenia (63%); however, approximately half of the patients persisted with dose adjustment.^[6]^ Second, in newborns with congenital CMV disease, oral administration of 16 mg/kg VGCV provided comparable plasma GCV concentrations to those achieved with intravenous administration of 6 mg/kg GCV.^[5]^ Finally, a phase III clinical trial that compared oral VGCV 16 mg/kg twice daily for 6 weeks with that for 6 months showed that the 6-month treatment group experienced a more effective and significant improvement in hearing and neurodevelopment than the 6-week treatment group.^[8]^ In the safety assessment, neutropenia was found to occur in approximately 20% of cases; however, the incidence in the 6-month treatment group did not exceed that in the 6-week treatment group.^[8]^ Therefore, in this clinical trial, VGCV was orally administered at a dose of 16 mg/kg twice per day for 6 months. The criteria for dose adjustment and drug withdrawal were set according to a phase III clinical trial by Kimberlin et al.^[8]^

Congenital CMV disease, which shows various symptoms and findings from birth, is a progressive disease. Because of the importance of evaluating changes in blood CMV loads and hearing and neurodevelopmental status before and after VGCV treatment during a test of its effectiveness, it is appropriate to design and conduct a randomized, double-blind, placebo-control clinical trial. However, the congenital CMV disease from birth, especially that involving the central nervous system, which is the subject of this clinical trial, should be treated ethically in the actual clinical setting, owing to evidence of the efficacy of VGCV treatment.^[8]^ Because it is difficult to ethically establish a placebo group, this study was conducted as an open-label, single-arm study containing only the drug-administered group.

Generally, a decrease in viral load is an index of the effectiveness of an antiviral drug when administered to treat viral infectious diseases. A phase III clinical study showed a relationship between high CMV load in whole blood before treatment and impaired cognitive and motor development at 24 months of age.^[8]^ In a Japanese study, VGCV was orally administered for 6 weeks to patients with congenital CMV disease who exhibited hearing impairment and the relationship between blood CMV loads and hearing abnormality at 1 year of age was evaluated.^[13]^ According to the findings,^[13]^ blood CMV load in a patient, who improved the hearing abnormality, declined to levels below the detection limit at 1 week after treatment initiation. However, for patients who still experienced hearing abnormality, blood CMV load detection was continued even in the treatment.^[13]^ Because reduction in CMV load is an indicator of clinical efficacy, the change in whole blood CMV load was set as the primary endpoint in our clinical trial.

An important sequela in patients with congenital CMV disease is hearing impairment. ABR is a hearing evaluation test that can be performed shortly after birth. In early phase III clinical trials in the United States, change in hearing status before and after treatment according to ABR was set as the primary endpoint. Therefore, the change in hearing status by ABR before and after treatment for 6 months is important in our clinical trial. Accordingly, this change was set as the important secondary endpoint.

To the best of our knowledge, this multicenter, open-label, single-arm study will be the first well-designed clinical trial to evaluate the efficacy of oral VGCV treatments in infants with congenital CMV diseases. The results of this trial will reveal the efficacy and safety of oral VGCV treatments and may aid in the approval of oral VGCV treatments for infants with congenital CMV disease by the government health insurance of Japan.

## Acknowledgments

This study is supported by the Kobe Clinical and Translational Research Center and National Center for Child Health and Development. The authors thank all staff for their involvement in this clinical trial.

## Author contributions

**Conceptualization:** Ichiro Morioka, Yasumasa Kakei, Akira Oka.

**Data curation and investigation:** Ichiro Morioka, Kazumichi Fujioka, Tetsushi Yoshikawa, Hiroyuki Moriuchi, Yoshinori Ito, Akira Oka.

**Formal analysis:** Takashi Omori.

**Methodology:** Ichiro Morioka, Yasumasa Kakei, Kandai Nozu, Kazumichi Fujioka, Tetsushi Yoshikawa, Hiroyuki Moriuchi, Yoshinori Ito, Akira Oka.

**Visualization:** Yasumasa Kakei.

**Writing – original draft:** Ichiro Morioka, Yasumasa Kakei, Takashi Omori

**Writing – review & editing:** Kandai Nozu, Kazumichi Fujioka, Tetsushi Yoshikawa, Hiroyuki Moriuchi, Yoshinori Ito, Akira Oka.

Ichiro Morioka orcid: 0000-0002-5685-2670.
